# Effect of LPS, dsRNA or interferons on the phagocytosis of dying cells or mycobacteria by macrophages

**DOI:** 10.1186/1753-6561-5-S1-P82

**Published:** 2011-01-10

**Authors:** Frederic Vanzembergh, Greet Brouckaert, Florence Nazé, Steven Van Gucht, Tom Vanden Berghe, Dmitri Krysko, Peter Vandenabeele, Michael Kalai

**Affiliations:** 1 Communicable and infectious diseases, Scientific Institute of Public Health, 1180 Brussels, Belgium; 2 Molecular Signaling and Cell Death Unit, Department for Molecular Biomedical Research, VIB and Department of Biomedical Molecular Biology, Ghent University, 9052 Zwijnaarde, Belgium

## 

Infection with intracellular pathogens can trigger a panel of innate immune responses including cell death. Coupled with phagocytosis this often leads to clearance of the invader. However, some pathogens use the process to disseminate and proliferate. During *Mycobacterium marinum* infection, dying infected macrophages recruit fresh ones to the site of granuloma formation. The recruited macrophages phagocytose infected cell remnants, get infected and die, thus ensuring efficient spread and multiplication of the pathogen. Maturation of granuloma, the characteristic lesions of tuberculosis, requires tumor necrosis factor (TNF) and interferon (IFN)-gamma and represents a stalemate between host and pathogen sufficient to arrest infection without eliminating the bacteria. Indeed, our macrophage / mycobacterial infection model demonstrates that virulent H37*RvMycobacterium tuberculosis* is more efficient in macrophage infection and killing than the attenuated *Mycobacterium bovis* BCG vaccine.

Today it is still unclear whether the signal promoting macrophage engagement in phagocytosis is originating from a pathogen or the infected host, and whether the effect is general or target specific. Therefore, we used a quantitative cell line based assay to study the effects of PAMPs or cellular alarm signals on the phagocytic engagement and capacity of macrophages to engulf virulent or attenuated mycobacteria and dying cells. Our results demonstrate that pretreatment of macrophage like cells with double stranded RNA, lipopolysaccharide, type I or II IFN but not with TNF, can significantly increase their capacity to phagocytose apoptotic and necrotic cells but has little effect on the phagocytosis of free mycobacteria. Although this macrophage activation process is probably an innate immune response reinforcing the capacity of the host to dispose of dying infected cells, pathogens may exploit it for their propagation.

